# Value of contrast-enhanced endoscopic ultrasound and elastography in the diagnosis and evaluation of portal vein thrombus

**DOI:** 10.1055/a-2783-4151

**Published:** 2026-02-13

**Authors:** Jun Li, Yingqun Zhou, Junshan Wang, Jiao Feng, Maochun Tang, Yilong Wang, Feng Liu

**Affiliations:** 1 278245 Department of Gastroenterology, Shanghai Tenth People’s Hospital, Tongji University School of Medicine, Shanghai, China; 2 654234 Department of Gastroenterology, Shanghai Tenth People’s Hospital Chongming Branch, Shanghai, China

**Fig. 1:**
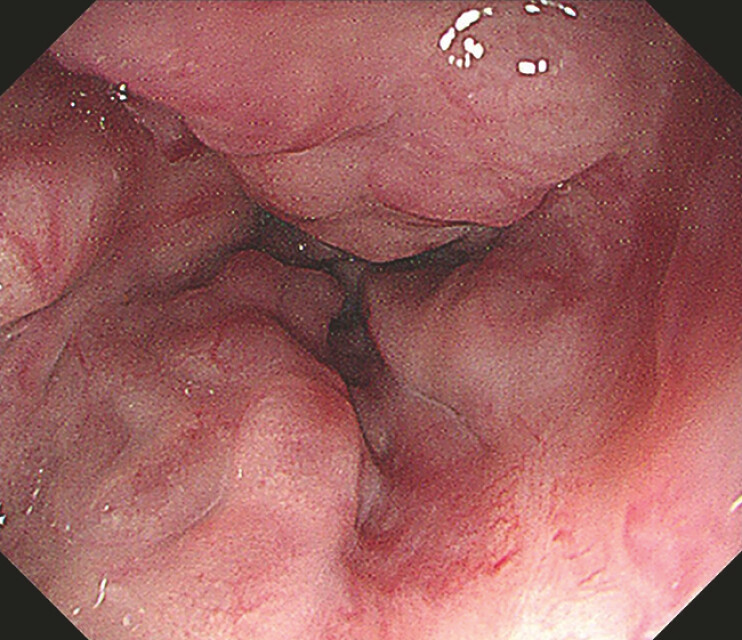
Gastroscopy showed progressive esophageal varices.

**Fig. 2:**
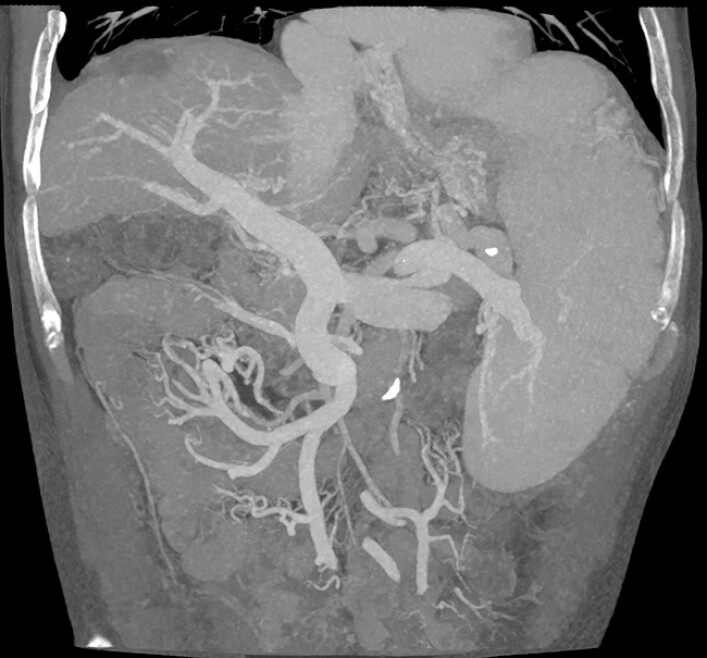
CT venography showed opacification of the main portal vein and right branch. CT, computed tomography.

**Fig. 3:**
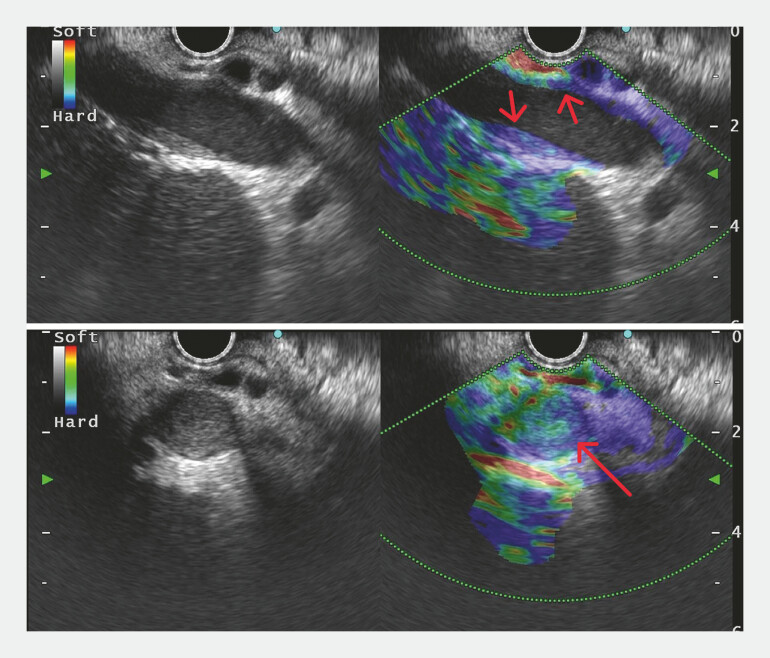
Elastography showed the thrombotic area appearing “blue” (red arrow).

**Fig. 4:**
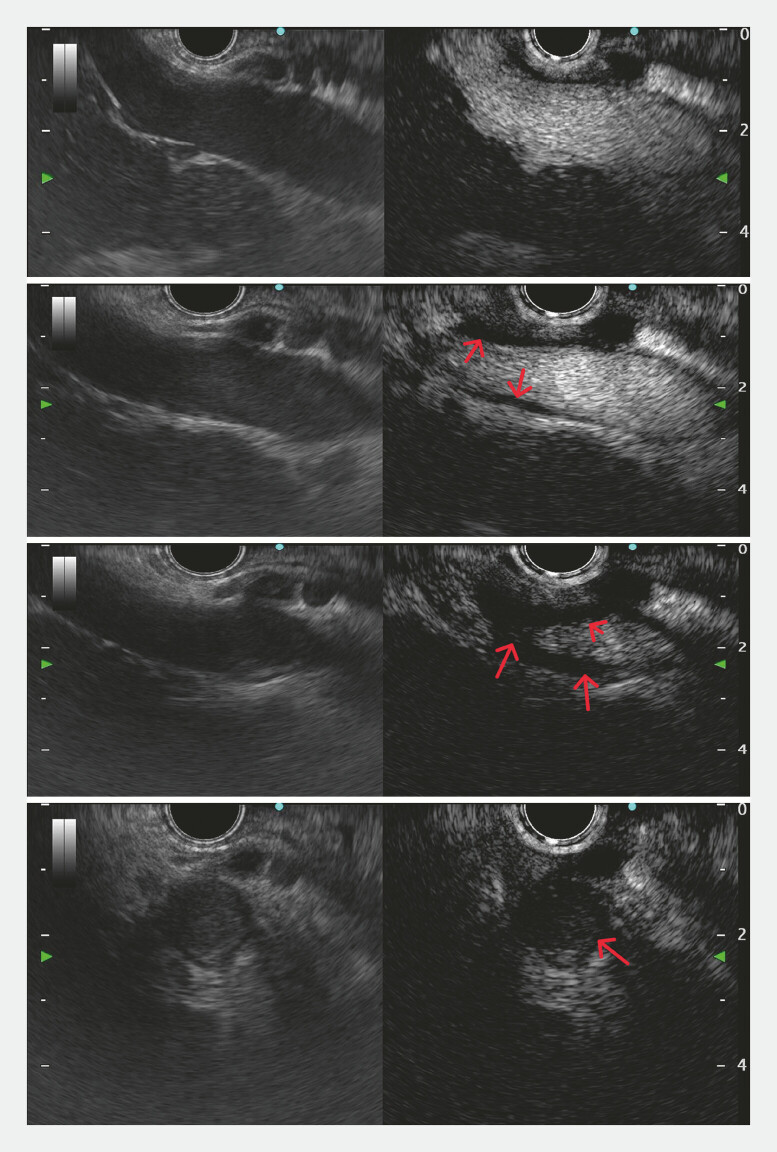
CEUS showed the demarcated thrombotic areas without internal enhancement from the non-thrombotic area (red arrow). CEUS, contrast-enhanced endoscopic ultrasound.

A 71-year-old man with hepatitis B virus-related cirrhosis and an 8-year history of hepatocellular carcinoma treated with repeated transcatheter arterial chemoembolization presented for the sequential management of gastroesophageal varices. Despite previous endoscopic interventions including glue injection and variceal ligation, follow-up gastroscopy showed progressive esophageal varices ( Fig. 1 ). Computed tomography venography revealed portal vein dilation with adequate contrast filling in the main portal vein and right branch, while the left branch showed stenosis/occlusion suggesting possible cavernous transformation ( Fig. 2 ). Endoscopic ultrasound (EUS) confirmed portal vein dilation (16.8 mm) with a reduced flow velocity (10.6 cm/s). In addition, EUS revealed a patchy mural thrombus in the main portal vein ( Video 1 ). Elastography revealed “blue” in the thrombotic area with strain ratio measured ( Fig. 3 , Video 1 ), indicating the stiff texture consistent with chronic thrombosis. Contrast-enhanced EUS (CEUS) with sulfur hexafluoride microbubbles revealed that the non-thrombotic region demonstrated patent blood flow with homogeneous enhancement, while the thrombotic area appeared sharply demarcated without internal enhancement ( Fig. 4 ). No arterial hyperenhancement or delayed “wash out” were observed in the thrombotic area, supporting the diagnosis of benign thrombosis. Following multidisciplinary discussion, the hepatic venous pressure gradient measurement was performed with a result of 23 mm Hg, leading to therapeutic decision of transjugular intrahepatic portosystemic shunt.

Contrast-enhanced endoscopic ultrasound and elastography in the diagnosis and evaluation of portal vein thrombus.Video 1

This case represents the first reported application of CEUS and EUS-elastography for portal vein thrombus (PVT) evaluation. While transabdominal CEUS has been described for PVT characterization and elastography for deep vein thrombosis 1 2 , the EUS approach provides a superior resolution due to its proximity to the portal vein, overcoming the limitations of body habitus and bowel gas. CEUS enables precise microvascular assessment crucial for malignancy exclusion, while elastography offers the quantitative stiffness measurement for thrombus aging. These techniques allow comprehensive thrombus characterization, informing therapeutic decisions and anticoagulation strategy in complex portal hypertension cases.

Endoscopy_UCTN_Code_TTT_1AS_2AG
